# The Poor Man's Home.—XXIII

**Published:** 1898-01-29

**Authors:** 


### Modern Sociology.

### THE POOR MAN'S HOME.—XXIII.

### Model Lodging-houses.

The dwellings we have hitherto spoken of have been intended for the occupation of a family. But those who are members of a family are not, in any class of society, the most to be pitied. The small earnings of two or three persons will keep all in comfort together, while they would not easily support any one singly. Also, among the poor as among the rich, the restraints of family life tend to keep the members of a household from many temptations, which are felt by the unattached. This is so well recognised that we have many religious and charitable associations which make it their chief work to establish communication with young persons of both sexes when they first leave their homes. Many young people, just entering the world, and exposed to temptation, but having no natural bias towards it, can be left to the agency of such associations; but there are others for whom such agencies can do nothing, and who, indeed, desire none of their good offices. Some of these people are admittedly disreputable; others are merely poor and lonely, but are too old or too independent to accept any help which seems to imply either guardianship or patronage. There are casual workers of all kinds who earn their bread in one town to-day and in another next week; there are old people who have no families, or whose children have migrated to some other place; and, lastly, there are tramps and vagrants who spend their lives wandering from place to place. All these live, not in homes, but in lodgings; and anything more miserable than the lodging-houses of the poor it would be difficult to conceive.

Suppose a young man, whose earnings allow him to pay not more than half-a-crown or three shillings a week for his lodging. If he lodges with a family in his own station, he will certainly not get a room to himself for that sum. He must share the room, and probably the bed of some member of the household, who may as often as not be a most undesirable companion. He has no privacy; he must be in every way one of the family, which even in the most favourable circumstances will be trying at times, and may involve him in all manner of unwelcome annoyances. But many of the lonely poor will not accept such family lodgings, and betake themselves to a regular lodging-house. Till recently, these lodging-houses have been objectionable in every way; huddling, discomfort, bad sanitation, and promiscuity have been their most marked characteristics; and they have been the scene of many quarrels, riots, and even bloodshed. For bedding there might be a filthy mattress, but as often only a sack spread on the floor. The number of occupants in any room was limited only by the amount of floor space, and as there is no separation of one bed—to honour it by that name—from another, the occupant must lie down with his clothes on, for fear that any one of his score of unknown companions is a thief, who will rob him in his sleep of any little valuable he may possess. In many of these places there was no adequate arrangement for the separation of the sexes. They were the homes of the most open and degraded immorality. From them also many dangerous epidemics have radiated, and, so many of the occupants being tramps, have been thus carried from town to town.

It was a recognition of these facts that caused the English legislature to undertake the regulation of common lodging-houses. The Public Health Act of 1875 ordains that all local sanitary authorities shall keep registers of the common lodging-houses situated within their districts, of the number of lodgers, and of the names and residences of the keepers. Before a house can be registered, and therefore before it can legally be used as a lodging-house, it must be inspected and approved by a competent sanitary official; and a certificate of character is demanded from any one who desires to keep it for that purpose. A sign declaring it to be a "registered common lodging-house" must be affixed in some conspicuous part of the outside of the building. The local sanitary authorities are also empowered to make regulations for the number of lodgers who may be kept in any house, and to provide for the separation of the sexes; to insist on proper cleanliness and ventilation; to arrange for giving notice in the case of infectious disease occurring in the house, and for preventing its spread; and, indeed, to take all possible measures to make such houses as good as may be, and to prevent their being a danger either to health or morals. The walls and ceilings of these houses must be whitewashed twice a year, and a penalty of £2 is inflicted for neglect of this precaution. If required to do so by the authorities, the keeper of the lodging-house must report the presence of beggars or vagrants; and the authorities have the right of entrance at all times. For disobeying any of these regulations, as well as for neglecting to report any cases of infectious disease which may occur, the keeper of a lodging-house may be fined £5. The Local Government Board has also prepared a set of rules for the guidance of sanitary authorities in their inspection of houses which are to be occupied as common lodging-houses, stating in detail the sanitary requirements to which a sanitary inspector should give his special attention in looking over the premises. He is of course expected to inquire carefully into the drainage of the house, and to see that walls, roofs, and floors are in good repair. He must note if the position of rooms, windows, and staircases is such as to permit of through ventilation. Rooms which have no chimneys must be provided with ventilating shafts. The bedrooms must not be used as kitchens or day-rooms. The papering of inside walls is not permitted. Each inmate must have 300 cubic feet of air-space allowed him, and a supply of ten gallons of water for each inmate per day is recommended, although not insisted on. The quality of the water must, however, be proved to be satisfactory. Closets or privies must be provided in the proportion of one to every 20 inmates. It is also recommended that washing accommodation should be provided outside the sleeping-rooms.

These regulations improved the sanitary condition of the poor man's lodging; but they did little towards making it a comfortable hotel, with as much convenience as the sums which the lodgers paid for the accommodation would permit. To undertake this has been a later development of philanthropy. It was first undertaken by the municipality of Glasgow, and proved such a commercial success as to justify private persons in following in the municipal footsteps.

